# Intramuscular autologous blood therapy - a systematic review of controlled trials

**DOI:** 10.1186/s12906-019-2643-0

**Published:** 2019-09-05

**Authors:** Katja Oomen-Welke, Roman Huber

**Affiliations:** 1 Center for Complementary Medicine, Institute for Infection Prevention and Hospital Epidemiology, University Medical Center Freiburg, Faculty of Medicine, University of Freiburg, Breisacher Str.115b, 79106 Freiburg, Germany; 2 Present Address: Todtmoos, Germany

**Keywords:** Complementary medicine, Whole blood, Immunomodulation, Urticaria

## Abstract

**Background:**

Autologous whole blood (AWB) is used in complementary medicine for the treatment of infections and skin disorders. So far, the efficacy of AWB is discussed controversially.

**Methods:**

To estimate the efficacy of AWB therapy and to gather evidence in regard to effector mechanisms, we effected a systematic review of articles accessible through Pubmed and Cambase. Further trials were identified through references and by contacting study authors. Prospective controlled trials concerning intramuscular AWB therapy were included with the exception of trials using oxygenated, UV radiated or heated blood. Information was extracted on the indication, design, additions to AWB and outcome. Full texts were screened for information about the effector mechanisms.

**Results:**

Eight trials suited our criteria. In three controlled trials about atopic dermatitis and urticaria, AWB therapy showed beneficial effects. In five randomized controlled trials (RCTs), two of which concerned respiratory tract infections, two urticaria and one ankylosing spondylitis, no efficacy could be found. A quantitative assessment was not possible due to the heterogeneity of the included studies. We only found four controlled trials with sample sizes bigger than 37 individuals per group. Only one study investigated the effector mechanisms of AWB.

**Conclusions:**

There is some evidence for efficacy of AWB therapy in urticaria patients and patients with atopic eczema. Firm conclusions can, however, not be drawn. We see a great need for further RCTs with adequate sample sizes and for investigation of the effector mechanisms of AWB therapy.

## Background

The idea of using blood for healing purposes is not a new one. In ancient cultures, blood was used for magical purposes including healing rituals. Autologous blood therapy has been used in European medicine since the end of the nineteenth century [1]. Having been gradually discarded from general medical use from the 1950s onwards [1], complementary medicine still preserves it as a method to enhance the immune system [2], treat dermatological complaints such as urticaria and atopic dermatitis [3], ease pain in tendons and joints [4], improve tissue repair and is therefore also used for wound healing and treating tendon problems [5]. In Japan, intramuscular autologous blood or serum injections have traditionally been used for chronic urticaria [6].

The effector mechanisms of autologous blood therapy are widely unclear. It is considered as a stimulation or regulation therapy, which implies that a bodily self-healing reaction is elicited by a counter-regulation to irritation [1]. Therefore, mild reactions such as local bruises and soreness in the injection area have been regarded as inherent to the therapy [1, 7].

There are different methods of applying autologous blood: intravenous injection [1], intramuscular injection [2], local injection e.g. in tendons, ligaments [4], joints [4, 8], conjunctivas [1], wounds [5, 9], and ulcers [10]. Furthermore, modifications such as the injection of autologous blood products have been derived from autologous blood therapy. One important modification of AWB is the injection of autologous serum (AS), which is supposed to be experienced as less painful by some patients and for which recent RCTs show beneficial effects in urticaria [11, 12]. In order not to mix up different methods we focused in our review on AWB, which is the original method used in complementary medicine [2–5].

Major side effects of AWB have not yet been noted. If the blood has not been manipulated before reinjection and normal hygiene standards regarding injections are being observed, the infection risk is low [7].

So far, the efficacy of autologous blood is discussed controversially [1]. In this review, we focus on one of the simplest application modes – the intramuscular injection of autologous whole blood with or without further additives. As physicians do not need any special equipment nor any particular training other than what is conveyed at medical school in order to apply this technique, it represents no further financial strain on the health care system nor on the medical professionals themselves.

To investigate the efficacy and effector mechanisms of AWB injections, we viewed prospective controlled trials examining intramuscular AWB therapy for information about efficacy and mode of action.

## Methods

### Inclusion and exclusion criteria

We sketched out a plan based on general criteria concerning systematic reviews. As the aim of the review is to assess the efficacy of intramuscular AWB therapy, all other application modes were predefined excluded. Since heating, oxygenation and UV radiation of AWB require special equipment, this modification of autologous blood therapy was, therefore, excluded. We also excluded trials about the administration of blood products other than AWB (e. g. autologous plasma or serum), as we intended to investigate the basic application mode of AWB that does not require special equipment other than syringe and needle.

We also excluded retrospective and uncontrolled trials, furthermore trials that had only been published as abstracts or medical convention reports.

Prospective controlled trials involving intramuscular AWB therapy with or without further additives examining all kinds of patient population and indications were included in this review.

### Information sources and search strategy

In April and May 2017 a systematic search was performed in Pubmed in English using the following terms: “autologous blood injection”, “autologous blood eczema”, “autologous blood dermatitis”, “autologous blood atopic”, “autologous blood urticaria”, and “autohemotherapy”. Additionally, Cambase was searched in April 2017 for the key word “autologous blood” in English and German. References of related articles were scanned irrespective of language and study authors contacted for further information. Trials with available full text were selected according to the inclusion and exclusion criteria.

### Data extraction

From every included trial information was extracted on:

1. the indication
2. if a sample size calculation had been made
3. sample sizes
4. dosage, duration and frequency of the intramuscular autologous blood injection
5. additions to AWB before injection
6. outcome
7. information on investigations concerning the effector mechanisms.

The risk of bias of the included studies was evaluated using Jadad-Score. Further risks of bias not contained in this score were assessed individually by screening the respective publications. A quantitative assessment was not possible due to the heterogeneity of the included studies. We therefore summarized the trials individually, presenting their study design, results and limitations.

Search, screening by title, abstract and full text, eligibility assessment, data extraction and presentation was effected by KOW under supervision of RH.

## Results

**Fig. 1:**
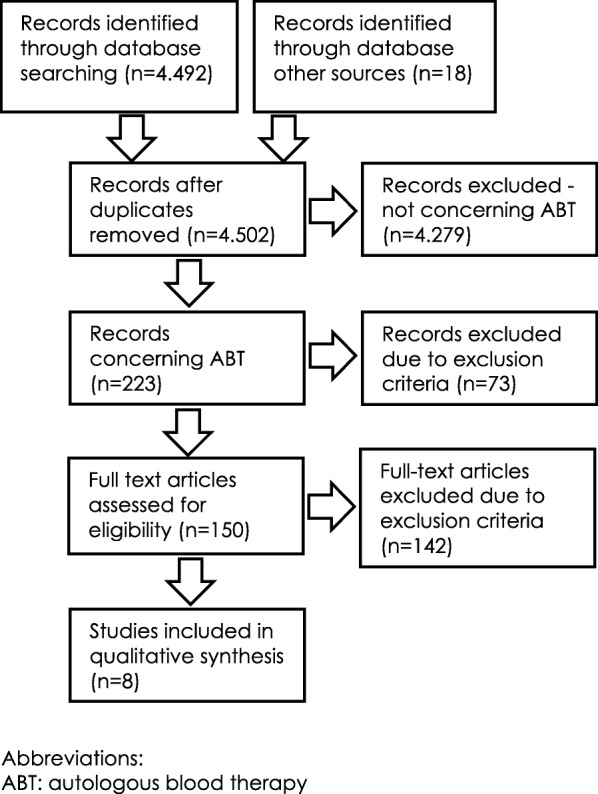
Flow diagram

The systematic search in Pubmed and Cambase provided 4.492 potentially relevant records. Other sources such as scanning references and contacting study authors led to 18 records. After screening titles and abstracts as well as removing duplicates, 223 records seemed to address the application of AWB and were therefore assessed and sorted according to the application mode of autologous blood. Seventy-three did not meet the inclusion criteria of intramuscular application of autologous blood or were excluded due to the exclusion criteria. After assessment of the full texts of the remaining 150 records, 142 were excluded due to exclusion criteria. Figure 1 shows the study selection process.

As a result, eight studies could be identified that suited the aim of this review. One of the trials had been conducted in the United Kingdom, one in Turkey, one in Egypt, one in China, and four in Germany. The publications had been released between 2000 and 2012 and concerned a total of 642 patients, 281 of whom had been treated with AWB. The times of administration of intramuscular AWB varied between three and ten injections once to thrice weekly, the dosage between one and five milliliters.

Four controlled trials were devoted to the treatment of urticaria. The other four RCTs concerned each one a different indication, including atopic dermatitis, ankylosing spondylitis, treatment of common cold and prevention of recurring respiratory tract infections. 

### Quality assessment

Four RCTs presented groups of ten to 25 patients, thus displaying characteristics of a pilot study, whereas the other four controlled trials showed somewhat larger dimensions with groups between 37 and 58 patients. However, only two of the latter were part of the three RCTs that had been double-blinded and therefore reached a Jadad-Score of 4 to 5 of 5. Two RCTs had been single-blinded and three controlled trials had not been blinded at all. The Jadad-Score evaluation is displayed in Table 1.

**Table 1:** Quality assessment controlled clinical trials according to the JADAD Score [13]

Author	Randomized	Randomisation appropriate	Double blind	Blinding appropriate	Description of withdrawals and dropouts	Total score
Abdallah et al. [14]	yes	yes	no	no	yes	3
Hensler, Gündling et al. [15]	yes	yes	yes	yes	yes	5
Jobst, Altiner et al. [2]	yes	yes	no	no	yes	3
Kocatürk et al. [16]	yes	no	no	no	yes	2
Min et al. [17]	no	no	no	no	no	0
Pittler, Ernst et al. [3]	yes	yes	yes	yes	yes	5
Schirmer, Fritz, Jäckel [18]	yes	yes	yes	no (not stated)	yes	4
Staubach et al. [19]	yes	yes	no	no	yes	3

Of all included trials, only Gündling et al. reported that they had calculated, but not reached, the required sample size of 70 individuals per group to obtain statistically reliable results [15].

### Efficacy of AWB

Five RCTs found no statistical difference between the groups, including four of the five larger RCTs. One of these, a non-blinded trial about the prevention of recurring respiratory tract infections [2], used as control instead of Placebo Engystol® (Biologische Heilmittel Heel GmbH, Baden-Baden, Germany), a homeopathic remedy made of sulfur and white swallow-wort (*Vincetoxicum hirundinaria*) which is used for the prevention of common cold and has antiviral properties in vitro [20]. As two treatments with possible but unclear efficacy were compared in this trial no conclusion on efficacy of AWB therapy can be drawn from it.

Another one of these RCTs compared AWB with autologous serum injections for chronic urticaria in 30 ASST-positive patients and found no statistical difference between the groups either [14]. The treatment was administered once weekly for 8 weeks (2,5 ml at the first, 5 ml in subsequent treatments). Outcome measure was the Urticarial Total Severity score (TSS). The difference between the mean TSS at baseline and in week 8 as well as week 12 was highly significant in both groups (*p* < 0,01). Because again two treatments with possible but unclear efficacy were compared in this trial no conclusion on efficacy of AWB therapy can be drawn from it.

Also, a RCT investigating AWB therapy in patients with ankylosing spondylitis found no significant difference between verum and control group [18]. In the double-blinded trial 1 ml AWB mixed with 1 ml *Formica rufa* 6x was injected intramuscularly twice weekly for 4 weeks. The control group was treated in the same frequency and application mode with 1.5 ml sodium chloride 0.9% solution. This trial was effected in a rehabilitation facility. All patients of both groups underwent in parallel an intensive therapeutic training scheme of 4 weeks. Both, verum and control group showed an improvement of symptoms. An additional effect of AWB mixed with *Formica rufa* could not be detected. The use of AWB and *Formica rufa* 6x injections as an adjunct to an effective therapeutic training program blurs the statistical analysis, so that efficacy of the injections cannot be estimated.

The fourth of the RCTs showing no statistical difference between verum and control group was effected by Gündling et al. about the treatment of common cold [15]. The trial was double-blinded, in the verum group 2 ml of AWB were administered thrice weekly. The control consisted in the application of the same amount of sodium chloride solution, also thrice weekly. Only 10% of the patients who were asked agreed to participate in the trial and the rate of discontinuing patients was high. Reasons for patients to refuse to participate might be due to the time-consuming treatment and the declination of intramuscular application of Placebo. The authors admit that these factors might have led to a selection of the patient sample. Otherwise, this study was well designed, reached the maximum Jadad score (Table 1) and was valid. Common cold can, therefore, not be regarded as promising indication for further studies with AWB therapy.

The last of the RCTs showing no statistical difference between AWB and Placebo group included 88 patients, but as these were split into six groups, the trial must still be considered within the scope of a pilot study [16]. Kocatürk et al. submitted patients with ASST-positive^1^ and ASST-negative urticaria to AWB, autologous serum and Placebo injections.

Three controlled trials reported a statistically significant effect of AWB compared with the control group. Pittler et al. presented a pilot RCT about efficacy of AWB injections in atopic dermatitis [3]. The study was well designed and showed beneficial effects in the verum group. However, the authors suggest that the treatment period of 5 weeks might not have been long enough to show the full potential of AWB injections, seeing as the improvement in the verum group was more pronounced towards the end of the treatment and especially towards at the end of week 9.

A larger dimensioned controlled trial [17] studied efficacy of AWB injections combined with traditional Chinese medicine and Western medicine in patients with chronic urticaria. One hundred fifty-seven patients were divided into three groups. One group was treated with traditional Chinese herbs, the other with Western medicine, consisting in antihistamines, vitamin c, and calcium. The third group received both traditional Chinese and Western medicine with additional AWB injections, diluted with sterile water and lidocaine, every 3 days for a period of 24 days in both buttocks. In the AWB group, 29 out of 52 patients were considered healed after the treatment, whereas in the TCM group 9 out of 52 and in the western medicine group 8 out of 53 showed no further symptoms. In the AWB group, only one out of 52 patients showed no improvement, whilst in the TCM group 13 out of 52 and in the Western medicine group 15 out of 53 patients did not report any change compared with baseline. As there was no control group combining TCM and Western medicine, the effectiveness of AWB injections cannot be estimated by this trial.

The third RCT with reported beneficial effects of AWB therapy was conducted single-blinded and Placebo controlled with sample sizes between 9 and 16 individuals per group comparing ASST-positive and ASST-negative urticaria patients [19]. The data showed a tendency for ASST-positive patients to benefit from AWB injections, whereas no difference between AWB and Placebo could be noted in ASST-negative patients. No significant differences in the level of FcεRI-targeting autoantibodies or of IgE could be noted between ASST-positive and ASST-negative patients, so that this study supplied no clue as to the effector mechanisms of AWB in urticaria.

Three of the four urticaria trials used the same dosage and frequency of AWB injections. Two RCTs with Placebo as control found a better response to AWB in ASST-positive patients [16, 19]. One small RCT [14] examined the difference in the efficacy of AWB and autologous serum in ASST-positive patients, which both showed equally good results.

All in all, four out of eight controlled trials used inadequate, that means potentially active controls (autologous serum injections [14], homeopathic injections [2], training schedules [18], Traditional Chinese Medicine, Western Medicine [17]). These study designs do not allow a statement about the efficacy of AWB, neither in the positive nor in the negative.

In the other four out of eight controlled trials, Placebos were used as controls. Two of the Placebo controlled trials showed no statistical difference [15, 16], while two others reported superior results of AWB [3, 19].

An overview of methods and results of all studies is displayed in Table 2.

**Table 2:** Results

Authors	Indication	Sample size	Design	Duration of treatment	Frequency and dosage	Outcome: doctor’s statement *p* = … in favor to patient’s statement	Comment
Abdallah et al. [14]	Chronic autoreactive urticaria	30 ASST+ (15 AWB, 15 AS)	RCT: AWB vs. AS, not blinded	8 weeks	1x/w; AWB: 1st injection 2.5 ml, following 5 ml; AS: 2 ml every time	AWB: *p* < 0.01 (weeks 8 + 12) AS: *p* < 0.01 (weeks 8 + 12) No statistical difference between treatments	
Hensler, Gündling et al. [15]	Treatment of Common Cold	114 (58 AWB, 56 Placebo)	RCT: AWB vs. Placebo double-blinded	1 week	3x/w 2 ml	No statistical difference	
Jobst, Altiner et al. [2]	prevention of recurring respiratory tract infections	75 (38 AWB, 37 Engystol®)	RCT: AWB vs. Engystol®, not blinded	5 weeks	2x/w 3 ml	No statistical difference between AWB and Engystol®	
Kocatürk et al. [16]	Urticaria	88 (ASST+ 59: 20 AWB, 20 AS, 19 Placebo; ASST- 29: 9 AWB, 10 AS, 10 Placebo)	RCT: AWB vs. AS vs. Placebo, single-blinded	10 weeks	1x/w; 1st injection 2.5 ml, following 5 ml	No statistical difference	
Min et al. [17]	Chronic Urticaria	157 (52 AWB, TCM (Chinese Herbs) + Western Medicine, 53 Western Medicine, 52 TCM)	Controlled trial: AWB + TCM + Western Medicine vs. TCM vs. Western Medicine, not randomized, not blinded	24 days	Every 3 days (total of 8 injections) 5 ml AWB + 1 ml lidocaine + 4 ml H2O, each side 5 ml	Treatment group (AWB + TCM + Western Medicine) more effective than the other groups (*p* < 0.05)	
Pittler, Ernst et al. [3]	Atopic Dermatitis	30 (15 AWB, 15 Placebo)	RCT: AWB vs. Placebo, double-blinded	5 weeks	1x/w; 1–2 – 3 – 2 – 1 ml	SASSAD score: week 5 *P* = 0.001 week 9 *P* < 0.001 patient’s statement: no statistical difference	
Schirmer, Fritz, Jäckel [18]	Spondylitis ankylosans	100 (51 AWB + Formica, 49 Placebo) + training scheme (both groups)	RCT: AWB + Formica vs. Placebo double-blinded	4 weeks	2x/w 1 ml AWB + 1 ml *Formica rufa* 6x	No statistical difference	Trial effected in rehabilitation facility
Staubach, Onnen et al. [19]	Chronic Urticaria	48 (29 ASST+: 13 AWB, 16 Placebo, 19 ASST-: 10 AWB, 9 Placebo)	RCT: AWB vs. Placebo, single-blinded	8 weeks	1x/w; 1st injection 2.5 ml, following 5 ml	ASST+ patients: AWB UAS −41%, *p* < 0.05, wheals duration + size −46%/−48%, *p* < 0.05, erythema −56%, *p* < 0.05, DLQI week8 + 70%, *p* < 0.005, week12 + 66%, *p* < 0.05, antihistamines −52%, *p* < 0.05; Placebo UAS −18%; ASST- patients: AWB UAS −21%, Placebo UAS −11%	

### Effector mechanisms

Only one of the studies included in this review made an approach to investigating the effector mechanisms of AWB therapy. Staubach et al. [19] determined anti-FCεRI expression in patients with urticaria and discovered that less than 20% of ASST-positive patients expressed anti-FCεRI. No significant differences in the level of FcεRI-targeting autoantibodies or of IgE could be noted between ASST-positive and ASST-negative patients, so that this study supplied no clue as to the effector mechanisms of AWB in urticaria. Furthermore, these results contradict previous reports that showed anti-FCεRI to be present in 40% of ASST-positive patients [19].

## Discussion

The studies included in this review show a great heterogeneity regarding study design, sample sizes, controls, dosage, frequency and duration of treatment as well as indications. For most indications, we found just one trial matching the inclusion criteria, except for urticaria, which is represented in four out of eight studies. This heterogeneity and the use of controls with uncertain activity made it impossible to give a quantitative résumé.

The quality of the studies varied as well. High quality was found in three of the eight controlled trials only [3, 15, 18]. Five studies had not been double-blinded [2] [14, 16, 17, 19] (moderate quality) and one was rated having poor quality [17]. The sample sizes in the studies mostly were small (four RCTs with < 37 per group) [3, 14, 16] [19] and the studies with higher sample sizes either used inadequate controls [2, 14, 17] or used three or more arms [16, 19], which made them in fact pilot studies. All in all there is some evidence for favorable effects of AWB-therapy in atopic eczema from one Placebo controlled RCT and in ASST-positive urticarial from two Placebo controlled RCTs.

A prior review on autohemotherapy from 2014 [7] was limited to urticaria and eczema, evaluated different interventions (autologous whole blood, but also autologous serum) and did not follow the Prisma guidelines. Four of the studies included in our review have also been analyzed in this previous review [3, 14, 16, 19], the conclusions regarding these studies were comparable to our review. Autologous whole blood and autologous serum injections were regarded as having similar effectiveness but this statement was based on limited evidence.

Regarding effector mechanisms some authors suggest that AWB is likely to have modulating effects on the immune function, e.g. on cytokines inducing activation of macrophages and T-cell-subpopulations [3], and might possibly desensitize patients against triggering factors including autoantibodies [21]. Others suppose that anti-idiotypes [19] might be induced and thereby the function of disease-inducing antibodies inhibited. In urticaria, as ASST-positive patients have been shown to express significantly less Th1 cytokines than normal controls, it was suggested that AWB might reduce Th2 cytokines and promote Th1 cytokines [19].

Except the one study included in our review mentioned above [19], we found two retrospective trials not included in our review that reported data concerning effector mechanisms [6, 22]. Mori and Hashimoto reported of an individual patient that ASST remained positive even after urticaria had been cured for several weeks [6]. The ASST is assumed to show the presence of autoantibodies against the high-affinity receptor for IgE (FCεRIα), which cause the release of histamine from mast cells and basophils [19]. This is why Mori and Hashimoto concluded that, at least in that one case, the effector mechanisms of AWB had to be others than inhibition of FCεRIα autoantibodies [6].

Olwin et al. [22] analyzed interferon-α, interferon-γ, and various cytokines in a cohort of 25 patients before the therapy as well as 24, 48 and 72 h after AWB therapy in patients with herpes zoster. They found that interleukin-4, interferon-α, and interferon-γ were higher than at baseline in the patients within 24 h after the AWB injection.

None of the findings regarding effector mechanisms have, however, been confirmed and it has, as a result of this review, up to now no convincing mode of action been shown for AWB-therapy. The fact that so far, the effector mechanisms could not be identified, is a limitation of intramuscular AWB therapy.

## Conclusions

Even though some trials report beneficial effects of intramuscular AWB therapy, the efficacy could not be ascertained. Therefore, further RCTs with higher quality and adequate sample sizes are required to clarify the efficacy of autologous blood therapy.

The effector mechanism of AWB has so far not been scientifically determined. Only one of the studies included in this review made an approach to investigating the effector mechanisms of AWB therapy [19], which leaves a gap that needs to be filled.

## Data Availability

The datasets used and/or analysed during the study are available from the corresponding author on reasonable request.

## Abbreviations

AS: Autologous serum
ASST: Autologous serum skin test
AWB: autologous whole blood
DLQI: Dermatology life quality index
RCT: Randomized controlled trial
TCM: Traditional Chinese medicine
UAS: Urticaria activity score

## Footnotes

1 ASST stands for Autologous serum skin test. The intracutaneous injection of autologous serum leads to an immediate hypersensitivity type skin reaction in some patients with urticaria [20].
